# Comparison of Pain Perception Between Local Infiltration and Inferior Alveolar Nerve Block Injection Techniques in Patients Undergoing Orthodontic Lower Premolar Extractions

**DOI:** 10.7759/cureus.48794

**Published:** 2023-11-14

**Authors:** Aditya H, Vinod K Krishna, Saravanan Lakshmanan, Murugesan Krishnan, Santhosh P Kumar

**Affiliations:** 1 Oral and Maxillofacial Surgery, Saveetha Dental College and Hospitals, Saveetha Institute of Medical and Technical Sciences, Saveetha University, Chennai, IND

**Keywords:** innovative technique, novel, premolar teeth, inferior alveolar nerve block, pain, local anesthesia, local infiltration, orthodontic extraction

## Abstract

Introduction

The most frequently used local anesthesia administration techniques for extraction of lower teeth in dentistry are local infiltration and inferior alveolar nerve block. Therapeutic extraction of premolars is the most common procedure done for patients undergoing orthodontic treatment. Inferior alveolar nerve block has been used most commonly for extraction of mandibular posterior teeth; however, it is a technique-sensitive procedure and has complications such as facial nerve palsy, trismus, and long duration of anesthesia. Local infiltration is a simple and effective technique for anesthetizing teeth prior to extraction.

Aim

This study aims to compare the efficacy of local anesthesia administered through inferior alveolar nerve block and local infiltration techniques for extraction of lower premolar teeth for orthodontic purposes.

Materials and methods

A prospective comparative study was conducted for a period of six months in which a total of 100 patients who were referred for extraction of lower premolar teeth for orthodontic purposes were included. Among 100 patients, 60 patients were males, and 40 patients were females with a mean age of 16.5 ± 1.25 years. The patients were equally divided into two groups, in which Group 1 received local infiltration and Group 2 received an inferior alveolar nerve block. The outcome parameters assessed were pain during injection and pain during extraction using the visual analog scale (VAS) score and Wong-Baker Faces Pain Rating Scale score. Statistical analysis was done using an independent sample t-test with SPSS version 23.0 software (IBM Corp., Armonk, NY) at p < 0.05 considered statistically significant.

Results

The difference in mean pain scores between the two groups during injection as assessed using the Faces Pain Rating Scale (p = 0.001) and VAS (p = 0.001) was statistically significant, with the infiltration group exhibiting less pain than the inferior alveolar nerve block group. The difference in mean pain scores between the two groups during extraction as assessed using the Faces Pain Rating Scale (p = 0.308) and VAS (p = 0.350) was statistically not significant, with the infiltration group not significantly differing from the inferior alveolar nerve block group. Thus, the pain during local infiltration was significantly lesser when compared to the inferior alveolar nerve block during injection, whereas pain perception during extraction was similar in patients with both injection techniques.

Conclusion

It can be concluded that local infiltration is less painful for the patient during injection and as efficacious as nerve block for extraction, hence local infiltration can be routinely used for lower premolar orthodontic extractions.

## Introduction

Local anesthesia is defined as “A temporary loss of sensation in a circumscribed area of the body. It is produced by an inhibition of the conduction process of the action potential in peripheral nerves without depressing the level of consciousness” [1]. Despite not anesthetizing the proprioceptive fibers of the affected nerves, profound local anesthesia completely eliminates pain, temperature, and touch sensations [2].

The most common difficulty faced by dentists is achieving profound anesthesia in patients undergoing extraction of teeth with pulpitis. An ideal dental extraction must be painless and comfortable for the patient. The painless procedure makes the patient develop a much more positive mentality for the proposed treatments. The topical anesthetics application prior to injection is the routine practice in dentistry, especially in pediatric patients. Therapeutic extraction of premolars is the most common procedure done for patients undergoing orthodontic treatment who are mostly young patients below the age of 25 years. Being young adults, the pain threshold is comparatively lesser in these patients. Thus, using the proper injection technique is essential to make the patient cooperate during extraction. The most often used injectable techniques for producing local anesthesia in these patients are infiltration and inferior alveolar nerve block (IANB) [3]. IANB has been used most commonly for the extraction of mandibular posterior teeth; however, it is a technique-sensitive procedure with complications such as facial nerve palsy, trismus, and long duration of anesthesia [4-6].

Due to various variables, including the difficulty in identifying landmarks, this procedure is challenging for beginners and has the greatest failure rates. Furthermore, it could lead to issues such as limited mouth opening, hematoma formation, and facial nerve palsy [7]. Mandibular infiltration has traditionally been avoided when treating mandibular posterior teeth because of its limited efficiency. This is likely due to the thickness of the buccal cortical plate, which prevents the local anesthetic solution from spreading [8]. Practicing clinicians have recently begun looking into the idea of making infiltration anesthesia a viable alternative to the IANB method [9]. The duration of IANB and the discomfort it causes from numb lips and tongue typically outlasts the duration of dental procedures, particularly extractions. Previous studies were done mainly comparing the molars extracted due to reasons like pulpitis or periodontitis but not for extraction of healthy premolar teeth as in orthodontic patients [6,7,10].

This study aims to compare the efficacy of local anesthesia administered through IANB and local infiltration techniques for the extraction of lower premolar teeth for orthodontic purposes. The objectives of the study were to assess and compare pain perception during injection and extraction among patients with local infiltration and IANB injection techniques.

## Materials and methods

Study design and setting

This prospective comparative study was performed in the Department of Oral and Maxillofacial Surgery, Saveetha Dental College, Chennai for a period of six months after obtaining approval from the Institutional Human Ethics Committee, Saveetha Dental College (approval number: IHEC/SDC/OMFS-2103/23/185). Written informed consent was obtained from all the study participants.

Inclusion criteria

All the patients irrespective of gender, with ages ranging from 12 to 25 years, referred for therapeutic extraction of lower premolars for orthodontic purposes were included in the study.

Exclusion criteria

Patients with existing systemic illness and referred for extraction of teeth other than premolar teeth were excluded from the study.

Sampling and recruitment

The sample size calculation was done with the help of G*Power software version 3.0 at 90% power. The sample size obtained was 96 and was rounded off to 100. A total of 100 patients between 12 and 25 years, referred for extraction of lower premolars for orthodontics purposes were divided into two groups of 50 each. Group 1 received the local infiltration injection technique (2 ml) and group 2 received the IANB injection technique (2 ml). The local anesthetic used was 2% lignocaine with 1:80,000 adrenaline (Xylocaine). Randomization was done with the help of an online random allocation software (RandomAlloc.exe, version 1.0). Details like the number of groups, participants, and anesthesia techniques used were fed into the software. Each participant received a random number. The treatment allocation number was placed in an opaque envelope. Allocation protocol was not available to the primary surgeon. Blinding was done by asking a secondary surgeon to give a nerve block or infiltration and extraction of the tooth was done by the primary surgeon to avoid bias. Effect size calculation was done using Cohen’s d formula for the two independent groups 1 and 2.

Procedure

**Figure 1:**
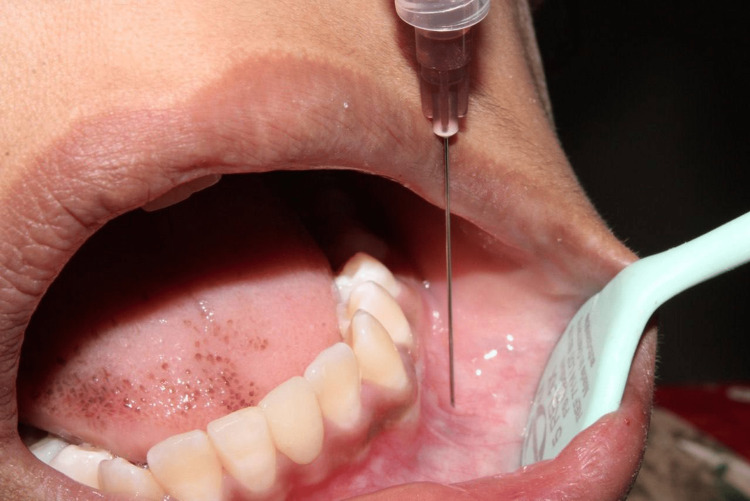
Local infiltration injection technique

**Figure 2:**
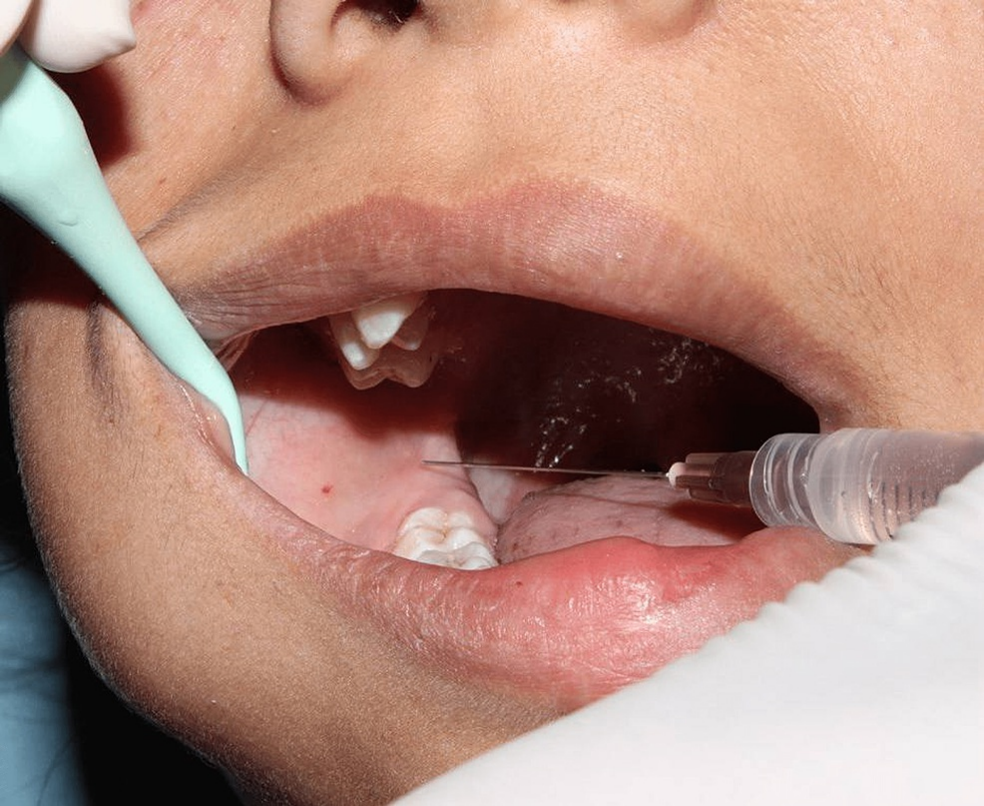
Inferior alveolar nerve block injection technique

Local infiltration (group 1) was given by injecting 2 ml of 2% lignocaine with 1:80,000 adrenaline (1 ml buccally and 1 ml lingually) at the mucobuccal fold (Figure 1). IANB (group 2) was given at the mucous membrane on the medial side of the mandibular ramus ¾th of anteroposterior distance from the coronoid notch at the deepest point in the pterygomandibular raphe by injecting 1.5 ml of 2% lignocaine with 1:80,000 adrenaline for inferior alveolar nerve and lingual nerve block and 0.5 ml was injected separately for long buccal nerve block (Figure 2). After confirming local anesthetic action, both objectively and subjectively, extraction of the lower premolars was done with the help of lower premolar forceps. Postoperative instructions and medications were given.

Outcome assessment

The outcome assessment was pain perception. It was assessed while giving injection and during extraction of the tooth using the Wong-Baker Faces Pain Rating Scale scores and visual analog scale (VAS) scores. Facial pain scale uses facial expression to evaluate the amount of pain. It has scores ranging from 0 to 10 with 0 as the least pain and 10 as the worst pain.

Statistical analysis

Statistical Package for Social Sciences (SPSS software, version 23.0; IBM Corp., Armonk, NY) was used to analyze the data. The normality of the results was assessed using the Shapiro-Wilk test of normality. The results were checked and found to follow a normal distribution. To comparatively analyze two different groups, an independent samples t-test was used. If the p-value was ≤ 0.05, the differences were considered statistically significant.

## Results

Among 100 patients, 60 patients were males and 40 patients were females, with a mean age of 16.5 ± 1.25 years. In Table 1, group 1 shows a mean value of 1.65 ± 1.321, while group 2 shows a mean value of 3.47 ± 1.555 for pain perception during local anesthesia administration assessed with the Wong-Baker Faces Pain Rating Scale. The difference between the mean values of the two groups was statistically significant (p-value = 0.001), with less pain exhibited in group 1 (local infiltration technique).

**Table 1:** Intergroup comparison of pain perception during local anesthesia administration using the Wong-Baker Faces Pain Rating Scale Group 1: local infiltration injection; Group 2: inferior alveolar nerve block injection; * statistically significant, independent samples t-test.

Group	N	Mean ± SD	P-value
Group 1	50	1.65 ± 1.32	0.001*
Group 2	50	3.47 ± 1.55	

In Table 2, group 1 shows a mean value of 1.71 ± 0.863, while group 2 shows a mean value of 3.93 ± 1.482 for pain perception during local anesthesia administration while using the VAS. The difference between the mean values of the two groups was statistically significant (p-value = 0.001), with less pain exhibited in group 1 (local infiltration technique).

**Table 2:** Intergroup comparison of pain perception during local anesthesia administration using the visual analog scale Group 1: local infiltration injection; Group 2: inferior alveolar nerve block injection; * statistically significant, independent samples t-test.

Group	N	Mean ± SD	P-value
Group 1	50	1.71 ± 0.86	0.001*
Group 2	50	3.93 ± 1.48	

Table 3 depicts the Wong-Baker Faces Pain Rating Scale score during extraction. Here, group 1 shows a mean value of 2.36 ± 1.362 while group 2 shows a mean value of 2.06 ± 1.370. The difference between the mean values of the two groups was statistically not significant (p-value = 0.308), hence pain perception during extraction was not significantly different between the two groups.

**Table 3:** Intergroup comparison of pain perception during extraction using the Wong-Baker Faces Pain Rating Scale Group 1: local infiltration injection; Group 2: inferior alveolar nerve block injection; * statistically significant, independent samples t-test.

Group	N	Mean ± SD	P-value
Group 1	50	2.36 ± 1.36	0.308
Group 2	50	2.06 ± 1.37	

Table 4 depicts the VAS score during extraction. Here, group 1 shows a mean value of 1.77 ± 1.117 while group 2 shows a mean value of 1.98 ± 1.009. The difference between the mean values of the two groups was statistically not significant (p-value = 0.350), hence pain perception during extraction was not significantly different between the two groups.

**Table 4:** Intergroup comparison of pain perception during extraction using the visual analog scale Group 1: local infiltration injection; Group 2: inferior alveolar nerve block injection; * statistically significant, independent samples t-test.

Group	N	Mean ± SD	P-value
Group 1	50	1.77 ± 1.11	0.350
Group 2	50	1.98 ± 1.00	

Based on our study results assessed with the help of the facial pain scale and VAS scores, at the time of injection, there was a significant difference between the two groups, with infiltration injection being less painful than IANB. However, during extraction, there was no significant difference between the two groups in pain perception. Cohen's d effect size calculation gave a value of 1.17, which signifies a large effect size in accordance with the results representing that infiltration is 1.17 times better than those with IANB.

## Discussion

Local anesthesia plays a critical role in dental treatment, especially for extractions where pain is inevitable. But to achieve proper anesthesia, the usage of appropriate techniques is essential. Though pain while injection can be managed with topical anesthetics, techniques like infiltration and nerve blocks require expertise. Despite being technique-sensitive and the possibility for complications, IANB still is considered the gold standard for the extraction of mandibular posterior teeth [11-13]. The possible complications are facial nerve palsy, reduction in mouth opening, and longer duration of anesthesia.

The reason for preferring IANB can also be attributed to the dense structure of the mandibular bone and the inability of local anesthesia to diffuse this structure via simple supra-periosteal infiltration [14]. However, there is literature evidence supporting that local infiltration of anesthesia could reach the tooth via multiple accessory foramina in the mandibular bone [14-16]. Hence, this study was conducted to determine the effectiveness of local infiltration and IANB for therapeutic extraction of lower premolars using the Wong-Baker Faces Pain Rating Scale and VAS scores. The systematic review by Tomlinson et al. states that the Wong-Baker Faces Pain Rating Scale is a rapid and simple scale with the psychometric qualities required to evaluate pain [17].

Our study results revealed that the facial pain scale score was significantly less during injection for the infiltration group than the IANB group, and there was no significant difference while doing extraction in both groups. A study by Bahrololoomi et al. revealed similar results in pain scores using the facial pain scale during infiltration and IANB [16]. On evaluation using VAS scores, the pain perception during IANB was significantly more than infiltration and while extraction of teeth there was no significant difference between the two injection techniques. Several studies found similar pain scores, with the infiltration technique exhibiting less pain than IANB. They reported during extraction additional local anesthesia was required only for a few patients in the infiltration group whereas for many patients in the IANB group [18,19]. They also reported that the onset and duration of anesthesia were longer in the IANB group than the infiltration group and mentioned that the infiltration injection technique, which has a lesser duration of action and faster onset of action is preferred for extraction of mandibular premolars [18-20].

According to Madeira et al., 87.3% to 96.2% of the specimens had accessory foramina in the human mandible [21]. Pogrel et al. state that the mental nerve branches and enters the lateral surface of the mandible [22]. Similarly, in our study, 80% of the patients were pain-free after giving local infiltration technique. Thus, based on the literature and our study results, it can be stated that the success of the local infiltration technique in the mandible may be attributed to the diffusion of local anesthesia through multiple accessory foramina in the bone. Therefore, considering all the side effects of IANB, an alternative method of simple infiltration technique can be chosen to safely replace IANB for mandibular posterior teeth (premolar) extractions.

Limitations of the study

The limitation of the study is less sample size, and the injection techniques were not compared with different local anesthetic agents. With multi-center study on a larger sample size, the study results can be generalized to the population and better reproducibility of results can be expected.

## Conclusions

The infiltration technique when compared to IANB is simpler with a shorter and sufficient anesthetic effect. Infiltration injections are easier to administer and more comfortable for patients. They can be utilized to avoid collateral innervations, achieve hemostasis when necessary, and shield the nerve trunks from harm. It can be concluded that local infiltration is less painful for the patient during injection and as efficacious as nerve block for extraction, hence local infiltration can be routinely used for lower premolar orthodontic extractions.
